# Patriotism and the Nurse-Training Schools

**Published:** 1915-10-30

**Authors:** 


### PATRIOTISM & THE NURSE-TRAINING SCHOOLS*

### II. THE LONDON HOSPITAL.

An account of the plans adopted at the London Hospital to ensure that its weighty responsibilities should be duly borne towards the sick poor of London no less than towards the nation at war serves two purposes. It illuminates the spirit of this training school in particular and of nurses in general. It serves to explain the mystery, which puzzles those most who know most about hospital work, of institution life proceeding uninterruptedly, while yet so large a proportion of skilled members of the staff have been spared for the service of the wounded.

### In the Early Days.

From the London Hospital on August 8 the first batch of nurses left for France. Their names are as follows:—Nurses Witherington, Carthew, Caven, Prichard, Grayson, Kempthorne, Daly, Wellbank, Heffernan, Clements, Deakin, Burrell, Garnett; Nurse Baillie to Chatham. By the end of August the following had gone also:—*France*: Nurses Dunk, Holmes, Cooke, Kewley, Ball, Walker, Hissey, Ashford, Lambert, Paulin, Dickson, Martini, Cooper, White, Wheatley, Sharpe, Goldthorpe, Phillips, Leacy, Crooks, Spicer, Hobson, Hutchinson. *Malta*: Nurse Farquharson *Egypt*: Nurses Berry, Stearn, Klamborowski. *Home Service*: Nurses James, Baillie, Webb, Fairbrother, Veitch, Flynn, Roberts, Reynolds, Sibley, Prettyman, Wood, Houlson, Black, Hall, Finch, Humphries. *H.S. "Oxfordshire"*: Nurse Webb.

Before the month of August was out the hospital, having set aside 250 beds, was receiving its first consignment of 300 British wounded, and up to the beginning of June 1915, 2,688 sick and wounded soldiers have been received for treatment. It will be seen, therefore, that the nurses who had the more difficult part to play of sitting still in the house were not debarred from the privilege of nursing the wounded. The work of training short-time probationers was at once put in hand, and the sudden influx of candidates of every social class, "clamouring to be received for short periods of training," added enormously to the labours of the authorities. In the seven months up to the end of July 1914 the numbers applying were just over 1,000; in the five months after the outbreak of war the numbers were 2,857, and this remarkable increase is well maintained during the present year. All applications had to be carefully dealt with, and as large a number as was possible without disorganising the wards were given opportunities for acquiring some knowledge of nursing. It is very satisfactory to learn that the matron would willingly have continued the training of almost all who came for three or six months, had they desired to remain to work for a certificate, and this seems entirely to refute the ill-natured charges made against the short-time candidate. Successive batches of R.A.M.C. orderlies have also been received since March for a month's training.

Drafts of London Hospital nurses have been dispatched during the past year at varying intervals, according to demands made by the Army and the Admiralty. Before war broke out the hospital had guaranteed to supply sixty nurses to the Admiralty and fifty to the Army. The latter undertaking has been more than doubled, while the Navy requirements have been less than estimated. We append the names and destinations of the nurses dispatched between September 1914 and October 1915:—*France*: Nurses Brothwell, Bond, Dodd, Wainwright, Loder, Buckingham, M. Hawkins, S. Hawkins, Williams, Ames, Nickalls, Turner, Barns, Wolsey, Davey, Firman, Gosset, Clancy, Bennet, Brodie, Riley, Haigh, Simmonds, Day, Richards, James, Allis, Linton, Wright, Barry, Mitchley. *Egypt*: Nurses Phillips, Adamson, Ferguson, Gammell, Parry, Selfe, Whitmarsh, Pearce. *Malta*: Nurses Williamson, Gawler, Cott, Race, Webb, Hack, Sherman, Leavold, Jeffery. *Gibraltar*: Nurses Paske and Sharp. *H.S. "Carisbrooke Castle"*: Nurses Honywood, Macleod, Marshall, Haines. By far the larger proportion, it will be seen, are working in France, Flanders, and Malta.

The following is a list of the nurses sent from the London Hospital to Q.A.R.N.N.S.R. during the war:—Nurses Webb, Leaver, Rudall, Edwards, Cave-Brown-Cave, Elvins, Hardcastle, Fox, Edwards, Rowland, Fox-Harvey, Sandison, Adams, Langridge, Muncaster, Bryant, McFie, Fraser, Mann, Williams. Ten other nurses, who have since resigned, were sent to the Naval and Naval Reserve Services. Five sisters left to take up work in Serbia against the wishes of the matron, but with this exception the loyalty with which the staff have accepted duty at home or abroad or in the institution according to requirements is a gratifying feature in the patriotism of this as of most other training schools.

### Former Members of the Staff.

Many former members of the nursing staff are doing distinguished work. Miss Becher, R.R.C. (formerly Sister Mellish), is Matron-in-Chief at the War Office. Miss McCarthy, R.R.C. (formerly Sister Sophia), is the principal matron in charge of the nursing arrangements in France. Mlle. Emilie Luigi is matron of the Hôpital Civil at Rheims, and on her has been conferred the decoration known as "Citée à l'Ordre du Jour," in recognition of her fine conduct in assuring the service of the hospital "with the greatest devotion and under constant danger during the German occupation and the bombardment."

The hearts of the whole world have been stirred with sorrow and indignation at the cruel execution in Brussels of another distinguished member of the London Hospital Training School, Miss Edith Louisa Cavell.

* The previous article appeared on Oct. 23.

